# Experimental Injury Rodent Models for Oropharyngeal Dysphagia

**DOI:** 10.3390/biology10050360

**Published:** 2021-04-23

**Authors:** Ji-Youn Kim

**Affiliations:** Department of Dental Hygiene, College of Health Science, Gachon University, Incheon 21936, Korea; hoho6434@gachon.ac.kr; Tel.: +82-32-820-4376

**Keywords:** oropharyngeal dysphagia, swallowing difficulty, experimentally induced dysphagia, rodent

## Abstract

**Simple Summary:**

Dysphagia is a symptom of difficulty in carrying food safely from the mouth to the stomach. Recently, the number of people complaining of discomfort in swallowing due to various causes increased. In order to develop an appropriate treatment for them, experimentally induced dysphagia rodent models that mimic human dysphagia have recently been developed. Therefore, in this study, the rodent models of oropharyngeal dysphagia reported so far was summarized. The article search was conducted using Medline, Embase, and Cochrane Library. For this study, 13 articles that reported an experimentally induced oropharyngeal dysphagia in rodents were selected. The following information was collected: injury type, animal type, induction protocol of dysphagia, main defects, and dysphagia screening. Advances in basic research using animal studies are paving the way for intensive research and active therapeutic strategies for patients with swallowing disorders. The information would be the basis for pre-clinical trials to overcome oropharyngeal dysphagia.

**Abstract:**

Oropharyngeal dysphagia is a disorder that can make swallowing difficult and reduce the quality of life. Recently, the number of patients with swallowing difficulty has been increasing; however, no comprehensive treatment for such patients has been developed. Various experimental animal models that mimic oropharyngeal dysphagia have been developed to identify appropriate treatments. This review aims to summarize the experimentally induced oropharyngeal dysphagia rodent models that can be used to provide a pathological basis for dysphagia. The selected studies were classified into those reporting dysphagia rodent models showing lingual paralysis by hypoglossal nerve injury, facial muscle paralysis by facial nerve injury, laryngeal paralysis by laryngeal and vagus nerve injury, and tongue dysfunction by irradiation of the head and neck regions. The animals used in each injury model, the injury method that induced dysphagia, the screening method for dysphagia, and the results are summarized. The use of appropriate animal models of dysphagia may provide adequate answers to biological questions. This review can help in selecting a dysphagia animal system tailored for the purpose of providing a possible solution to overcome dysphagia.

## 1. Introduction

### 1.1. Normal and Abnormal Swallowing

Food intake is the most basic requirement in humans and is essential for survival. Swallowing is a complex process involving more than 30 nerves and muscles [1]. The trigeminal, facial, glossopharyngeal, vagus, and hypoglossal nerves innervate the major muscles related to swallowing [2,3,4]. The coordination of these nerves and muscles can lead to normal and harmonious swallowing [2]. Key aspects of normal swallowing include carrying the food from the mouth to the stomach and protecting the airway. The swallowing process can be divided into oral, pharyngeal, and esophageal phases [1,2]. Diagnosis of dysphagia is generally classified into two types: oropharyngeal dysphagia, relating to a neurological problem in the mouth, larynx, and pharynx and esophageal dysphagia, relating to a physical problem in the esophagus such as a blockage or irritation. Oropharyngeal dysphagia is more prevalent and more severe than esophageal dysphagia [2]. Oropharyngeal dysphagia is a very common condition in elderly, neurological or neurodegenerative patients, and patients with head and neck diseases. It is associated with neural damage controlling swallowing, reduced pharyngeal sensitivity, and impaired efferent nerve or muscle drive. Impairments of the tongue, lips, or cheek can impede oral phase in the swallowing process. Weakness of the lip and cheek muscles can cause accumulation in the oral vestibule. Tongue dysfunction can cause impairments in mastication and the formation and transport of bolus. These symptoms generally result from tongue weakness, or coordination or sensory impairment [1,2]. Pharyngeal phase involves rapid sequential activities that play an important role in transporting the bolus through the pharynx to the esophagus. Weakness of the tongue base and pharyngeal constrictor muscles can cause bolus retention in the pharynx and its entry into the airway [1]. Swallowing and breathing are intimately coordinated [5,6,7]. During swallowing, temporary interruption of respiration is seen due to neural suppression of respiration, as well as structural closure of the airway by the movement of the oropharyngeal muscles, such as the soft palate and epiglottis [6]. It implies that oropharyngeal dysphagia can cause nutritional and respiratory complications.

### 1.2. Injury Animal Models for Dysphagia Studies

Understanding neuromuscular control related to swallowing can be confirmed through nerve stimulation and lesions in animal work. Stimulation studies can provide information on how specific nerves control swallowing [8,9]. Lesion studies can mimic injuries in humans and investigate the necessity of a brain region for a specific behavior [10]. Lesion studies serves as a bridge between basic science and behavior in the clinic. Studies of animals with experimentally induced lesions have provided important insights into the neural basis of behavior [10]. The data from human subjects are incomplete compared to animal work due to ethical limitations. Animal studies can provide clear and uncomplicated research opportunities and an insightful information to understand dysphagia. In particular, injury or surgical models for dysphagia studies are being developed and validated for examining dysphagia [11,12,13,14,15,16,17,18,19,20]. In these animal models, rodents are commonly used to investigate mechanisms underlying nerve or muscle injury, regeneration, and treatment. Recently, rodent models of oropharyngeal dysphagia were developed by inducing nerve injury in the tongue and facial muscles or impeding the movement of the pharynx [12,13,14,15,16,17,18,19,20]. Injuries associated with dysphagia, such as branch injury of the hypoglossal nerve, facial nerve, vagus nerve, recurrent or superior laryngeal nerve, or other sensory deficits can be replicated relatively easily in animal models. In addition, dysphagia can also develop as a complication of head and neck surgery or radiation treatment, and a representative rodent model has been developed [21,22,23]. Oropharyngeal dysphagia due to nerve and muscle dysfunction can occur owing to various underlying causes. For a comprehensive treatment of dysphagia, the exact cause must be identified, and cause-specific interventions must be provided to the patient. Therefore, this review aimed to provide a methodological summary of experimental induction and screening of oropharyngeal dysphagia in rodent models and to help researchers choose an appropriate animal model.

## 2. Methods

**Figure 1:**
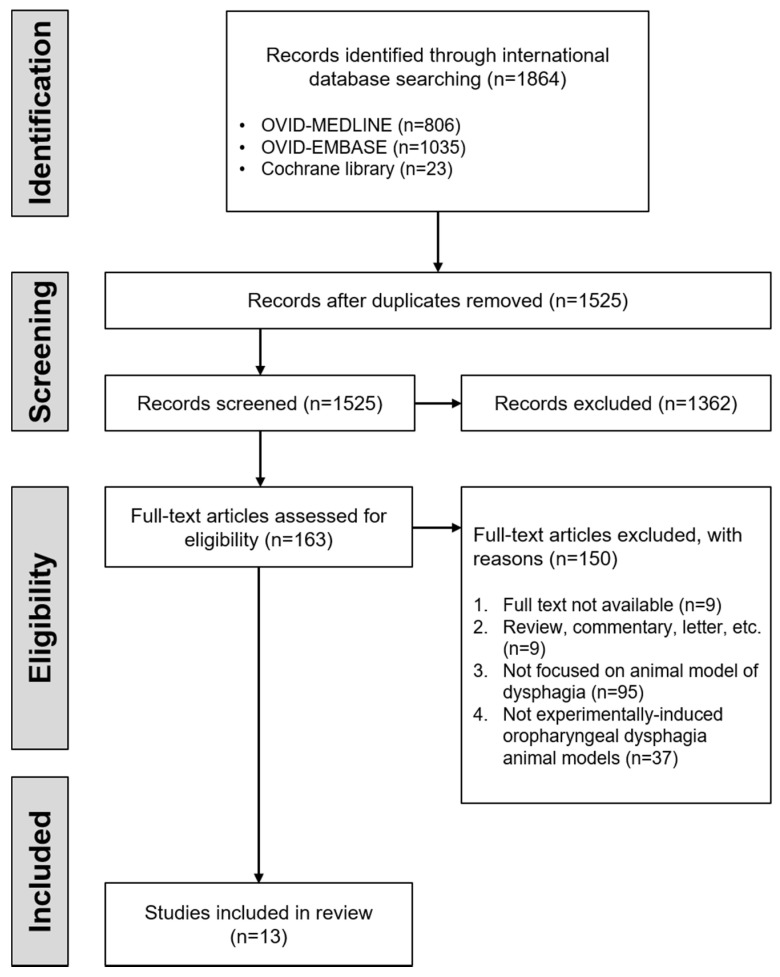
Flow diagram for literature searching and selection.

The literature search was conducted in March 2020 using Medline, Embase, and Cochrane Library. Our search criteria were established to be specific for the experimental studies using rodent models of dysphagia. The following terms were included in the search: “rodent”, “mice”, “rats”, “murine”, “mus musculus”, “deglutition disorder”, “dysphagia*”, “swallowing disorder*”, “swallowing difficult*”, “swallowing dysfunction”, “swallowing disease”, “deglutition disorder*”, “deglutition difficult*”, “deglutition dysfunction”, and “deglutition disease”. There was no restriction on the year of publication. In addition, only experimental studies using rodents have been selected. The following inclusion criteria were established: experimental animal models with induced oropharyngeal dysphagia and studies that evaluated experimentally induced oropharyngeal dysphagia. Reports regarding dysphagia due to aging and neurologic diseases were excluded because the purpose was to summarize the experimentally induced oropharyngeal dysphagia rodent models. Further, articles reporting the use of an animal model of dysphagia were excluded unless an evaluation for dysphagia was provided. Only peer-reviewed research papers were included in this study. All other studies were excluded from the analysis. Finally, a total of 13 articles were included for this review, as shown in Figure 1. To summarize the rodent models of oropharyngeal dysphagia induced by injury, the following information was considered: injury type, animal type, induction protocol of dysphagia, main defects, and dysphagia screening. Although the selected literature reported the outcomes of any treatment in an animal model of dysphagia, this review focused only on the information related to dysphagia.

## 3. Results

**Figure 2:**
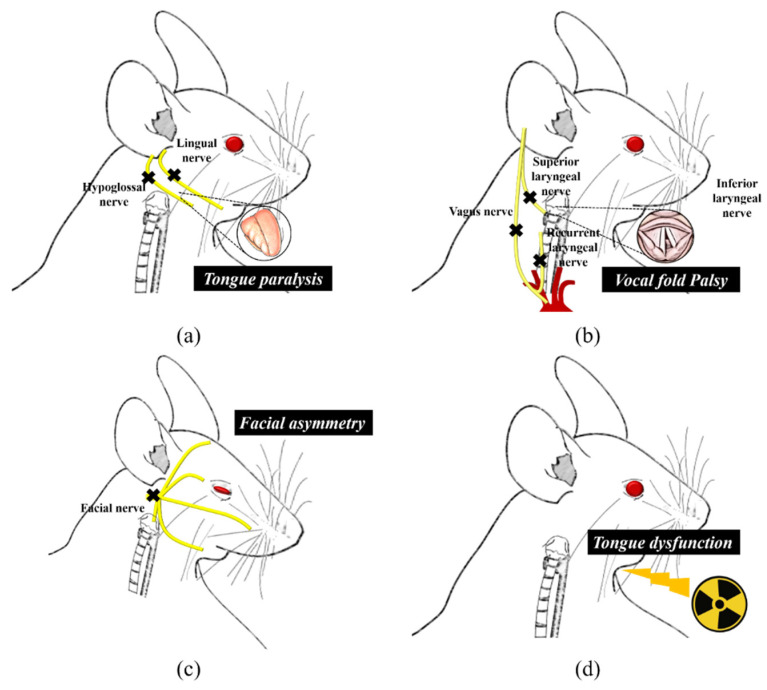
Rodent model of oropharyngeal dysphagia caused by various injuries. (**a**) unilateral lingual paralysis by hypoglossal nerve injury, (**b**) vocal fold paralysis by laryngeal or vagus nerve injury, (**c**) facial palsy by facial nerve injury, (**d**) tongue dysfunction by irradiation of head and neck regions.

Oropharyngeal dysphagia was induced by hypoglossal or facial or laryngeal or vagus nerve injury, radiation injury, gestational retinoid exposure injury in rodent models (Figure 2).

### 3.1. Lingual and Facial Muscles Injury

There was an animal model of oropharyngeal dysphagia with hypoglossal nerve injury expressing unilateral lingual paralysis [12,13,14] (Table 1). The animal model of oropharyngeal dysphagia was developed through unilateral section of the hypoglossal nerve in 250–300 g male rats. Dysphagia was verified through barometric plethysmography, water and food consumption, body weight measurement, and the physical exam of the tongue. Injury rat model with unilateral section of hypoglossal nerve led to swallowing deficit and drooling. Lingual paralysis as in this model includes the drooling problem, as well as lack of pharyngeal propulsion. The respiratory rhythm and the ventilator drive were decreased during swallowing [12,13]. In the case of both the hypoglossal nerve and lingual nerve being sectioned, water and food consumption decreased remarkably and significantly less weight was observed throughout the experimental period. The evidence of tongue bite was also observed [14]. There was the mouse model of oropharyngeal dysphagia with facial nerve injury [15]. Dysphagia was caused by sectioning the main trunk of the facial nerves of 6 to 12-months-old male and female mice. The VFSS results showed that dysphagia was caused while drinking and eating.

### 3.2. Laryngeal Injury

Animal models of oropharyngeal dysphagia with recurrent laryngeal nerve (RLN) or superior laryngeal nerve (SLN) injury were also reported [16,17] (Table 2). The models used 3 to 12-months-old male and female mice [16] and 9 to 10-weeks-old rats [17]. Oropharyngeal dysphagia was evaluated through laryngoscopy, VFSS, food consumption and body weight measurement, analysis of laryngeal residue. Unilateral RNL injury resulted in chronic vocal fold immobility but only acute dysphagia at the 1-week post-surgery. Bilateral RLN caused intraoperative asphyxiation and death. No evidence of dysphagia was observed in rodents with unilateral SNL injury, and dysphagia was confirmed only in rodents with bilateral SNL injury with decreased body weight and change in swallowing pattern and reflex [16,17]. In the rat model of oropharyngeal dysphagia with inferior laryngeal nerve (ILN) injury, unilateral vocal cord paralysis was observed while the swallow frequency and swallowing characteristics based on ventilation did not change [18]. Changed swallowing and ventilation coordination were observed in the oropharyngeal dysphagia rat model with oropharyngeal anesthesia by injecting 5% lidocaine orally [18]. There is a rat model of oropharyngeal dysphagia with unilateral cervical vagotomy. With male rats, a unilateral laryngeal paralysis was caused by a right or left cervical vagotomy under the superior laryngeal nerve and above the inferior laryngeal nerve [19,20]. Unilateral cervical vagotomy led to bronchial aspirations, unilateral vocal cord paralysis, and it was reported that chronic aspiration modifies respiratory pattern while swallowing.

### 3.3. Radiation Injury

Animal models of radiation injury dysphagia were developed in three methods: concurrent chemoradiation therapy (CCRT) injury; cyroinjuries that induces similar muscle damage to radiation injury; and direct irradiation method [21,22,23] (Table 3). After CCRT on the head and neck of rats, the mobility and strength of their tongue were assessed [21]. The strength of the tongue was weakened, and dislocation of the tongue was increased. When bilateral cyroinjuries were applied to the mylohyoid muscle of rats, bolus size and swallowing rate were decreased and licking patterns were changed [22]. When radiation injury was applied to tongue muscle, the strength of tongue and the contraction rate of tongue muscles were decreased [23].

### 3.4. Others

An animal model of dysphagia was built through gestational exposure to all-trans-retinoic acid (RA) daily on gestational days (GDs) 11 through to 13 [24] (Table 4). RA is a metabolic derivative of vitamin A, which acts as a signaling molecule that regulates the early embryonic development [25]. Dysphagia was verified by confirming that RA-exposed neonates could not swallow milk and confirming the empty stomach through autopsy.

## 4. Discussion

Swallowing food is a primary need in humans and a great pleasure. Most people can swallow without problems. This behavior is a fundamental activity observed even in animals and fetuses. Thus, eating disorders can greatly reduce the quality of life. The tongue and facial muscles play a central role in sucking, mastication, and swallowing. They play multiple roles in breaking down food and mixing it with saliva to form a bolus. The movement of the tongue is controlled by the hypoglossal nerve, and the facial muscles are controlled by the facial nerve. Any damage to them causes paralysis and renders the tongue and facial muscles incapable of functioning properly. Injury to the hypoglossal or facial nerve can occur because of infection, drug toxicity, tumor compression, trauma, and iatrogenic or unknown causes [15,26,27]. Injury to the hypoglossal or facial nerve is one of the most commonly observed cranial nerve injuries [26,27]. Rodent models of oropharyngeal dysphagia have been developed by sectioning the unilateral hypoglossal nerve [12,13,14]. Transection of the unilateral hypoglossal nerve led to unilateral lingual paralysis, which caused swallowing problems and drooling. In addition, the researchers assessed the relationship between lingual deficit and swallowing and breathing coordination. Using ventilation analysis, it was reported that unilateral lingual paralysis caused by transection of the hypoglossal nerve affected swallowing and breathing coordination [12,13]. Moreover, it was observed that the transection of both the hypoglossal (motor) and lingual (sensory) nerves caused lowering of water and food consumption and weight loss, which was more severe when compared to the effects of hypoglossal nerve transection alone [14]. A mouse model of oropharyngeal dysphagia was developed by surgically transecting the main trunk of the facial nerve. This resulted in oropharyngeal dysphagia in mice, with changes in lick and swallowing patterns during drinking and eating [15]. It was suggested that this injury mouse model could be used to study human dysphagia following facial nerve injury caused by various iatrogenic and idiopathic reasons.

Laryngeal paralysis refers to a phenomenon in which one or both vocal cords do not move because of abnormal neural control of the laryngeal muscles. The larynx plays an important role in swallowing by protecting the airway through reflexes. Therefore, laryngeal paralysis can lead to severe life-threatening complications. Two branches of the vagus nerve viz. superior laryngeal nerve (SLN) and recurrent laryngeal nerve (RLN) innervate the laryngeal muscles. The SLN runs towards the larynx and is divided into internal and external branches. The internal branch (sensory) pierces the thyrohyoid membrane and is distributed to the upper larynx. The external branch (motor) innervates the cricothyroid muscles. The RLN innervates all intrinsic muscles of the larynx, except for the cricothyroid muscles. The terminal branch of the RLN is called the inferior laryngeal nerve [28]. Ninety percent of laryngeal paralysis cases are peripheral paralysis, such as high vagal paralysis, low vagal paralysis, and SLN paralysis [29,30]. High vagal paralysis leads to paralyzed SLN and RLN. In low vagal paralysis, only the RLN is paralyzed. Most vocal cord paralysis is caused by RLN paralysis [31]. Because the SLN is intact, sensation in the larynx remains and the cricothyroid muscle is not paralyzed. SLN paralysis can occur after surgery, such as thyroidectomy, and is an uncommon lesion. In rodents, dysphagia was evaluated by developing the RLN or SLN injury model. Unilateral RLN transection or crush injury in mice led to vocal fold immobility and dysphagia. Bilateral SLN injury from transection or vascular clips also presents with obvious dysphagia in rodents [16,17]. Although changes in the coordination between swallowing and ventilation were not observed in the rat model with unilateral laryngeal paralysis induced by sectioning unilateral ILN, changed coordination between swallowing and ventilation was reported in a dysphagia rat model with induced oropharyngeal anesthesia by oral administration of 5% lidocaine [18]. A study has reported that there was chronic aspiration in the unilateral cervical vagotomy rat model induced by sectioning of the vagus nerve at a point under the SLN and above the ILN, which changed the breathing pattern while swallowing [19,20].

In another animal model, dysphagia was noted post the treatment of the oral and maxillofacial areas [32]. The cancer treatment paradigm of the head and neck has recently changed to concurrent chemoradiation therapy (CCRT), which is as efficient as the surgical procedure while avoiding tissue resection. Therefore, Benedict et al. developed a rat model of head and neck CCRT to evaluate the effect of CCRT on tongue function and structure. They proposed that the probability of dysphagia showing the strength of the tongue was decreased and displacement of the tongue was increased after CCRT [21]. Moreover, muscle damage is a frequent side effect of radiation therapy for head and neck cancers. A rat model of oropharyngeal dysphagia was developed by inducing damage to the mylohyoid muscle, which plays an important role in normal swallowing [22]. Tissue damage similar to that of radiation damage was caused by the cryoinjury method used for mylohyoid muscle damage. It was shown that the damage from the mylohyoid was sufficient to induce a change in deglutition. Cryoinjury is a commonly used method to induce muscle fibrosis in the heart [33,34], hindlimbs [35], and other organs [36,37]. Another report proposed the possibility of developing a dysphagia animal model via radiation-induced damage by showing functional changes in extrinsic tongue muscles, such as decreased strength and speed, after exposing the ventral side of the head and neck of the rats to radiation [23].

Finally, retinoic acid exposure at days 11, 12, and 13 of pregnancy in rats exhibited serious damage in swallowing ability [24]. However, due to high early fatality rate, it seems unlikely that this model can be used as an animal model of dysphagia.

## 5. Conclusions

Animal models mimicking human swallowing difficulties have been developed. Among the experimental animals, the rodent model has been regarded as a good candidate not only because it is morphologically similar to humans, but also because of the convenience in manipulation. Establishing and using experimental animal models that more accurately mimic the human pathological conditions is necessary. Therefore, this review summarized the rodent models of oropharyngeal dysphagia induced by various injuries. The information presented in this review will provide a great opportunity for researchers to select appropriate animal models and advance the understanding of the mechanisms of dysphagia.

## Figures and Tables

**Table 1:** Characterization of oropharyngeal dysphagia in rodent models of lingual and facial muscles injury.

Injury	Author (Year)	Animal/Sex, Age or BW	Method Inducing Dysphagia	Main Damage	Dysphagia Screening	
					Evaluation	Result
**Hypoglossal nerve injury**	Desombreset al. (2017) [12]	Rat/male, 250–374 g	Unilateral section of the hypoglossal nerve	Unilateral lingual paralysis	Barometric plethysmography	Sectioning of the right hypoglossal nerve achieved unilateral lingual paralysis, which caused swallowing deficit and drooling.
	Ouahchi et al. (2012) [13]	Rat/male, 250–350 g	Unilateral section of the hypoglossal nerve	Lingual motor deficits	Barometric plethysmography	Sectioning of the hypoglossal nerve led to a swallowing deficit and drooling. Respiratory rhythm and ventilatory drive during swallowing decreased after hypoglossal nerve sectioning but were unaffected during rest without swallowing, while swallowing rate and occurrence during respiratory cycle phases did not change.
	Doyleet al. (2016) [14]	Rat/male, 250–350 g	Transection of the hypoglossal nerve or hypoglossal and lingual nerve.	A combined functional impact on swallowing of tongue sensory (lingual n.) and motor (hypoglossal n.) loss	Measurement of water and food consumption and BW Physical exam of the tongue	The hypoglossal and lingual transection group consumed significantly less water and food than anesthetic, sham surgery, and hypoglossal transection group during PODs 1 to 3. The BW of the hypoglossal and lingual transection group was significantly lower at all postsurgical time points. A deviation to the right was observed in both the hypoglossal transection and hypoglossal and lingual transection groups during tongue protrusion. A substantial subset of animals within the hypoglossal and lingual transection group bit their tongues during PODs 1 to 5.
**Facial nerve injury**	Welby et al. (2020) [15]	Mouse/male and female, 6–12 months old	Transection of the main trunk of the facial nerve	Facial asymmetry, synkinesis, ocular sequelae, dysphagia	VFSS	Main trunk of facial nerve transection resulted in significantly slower lick and swallow rates during drinking and significantly slower swallow rates and longer inter-swallow intervals during eating.

BW, body weight; POD, postoperative day; VFSS, videofluoroscopic swallow study.

**Table 2:** Characterization of oropharyngeal dysphagia in rodent models of laryngeal injury.

Injury	Author (Year)	Animal/Sex, Age or BW	Method Inducing Dysphagia	Main Damage	Dysphagia Screening	
					Evaluation	Result
**RLN or SLN or ILN injury**	Mok et al. (2019) [16]	Mouse/male and female, 3–12 months old	RNL transection (unilateral and bilateral), SLN transection (unilateral and bilateral)	Ipsilateral VF immobility, dysphagia, dysphonia, dyspnea	Laryngoscopy (VF mobility) VFSS Histology	Unilateral RLN injury resulted in chronic VF immobility but only acute dysphagia. Bilateral RLN injury caused intraoperative asphyxiation and death. VF mobility was unaffected by SLN transection (unilateral or bilateral), and dysphagia was evident only after bilateral SLN transection.
	Tsuruta et al. (2018) [17]	Rat/male, 300–330 g, 9–10 weeks old	Bilateral SLN injury using a vascular clip	Swallowing difficulty	Measurement of food consumption and BW Swallowing analysis Measurement of the swallowing reflex Analysis of laryngeal residue	SLN injury animal model exhibited weight loss and drinking behavior changes. Following the SLN lesion, the number of interruptions during swallowing increased and the lick rate was reduced. The SLN lesion caused a delay in the onset of the swallowing reflex and gain of laryngeal residue in the pharynx.
	Ouahchi et al. (2011) [18]	Rat/male and female 2–3 months, 290–350 g	Unilateral laryngeal paralysis: 1) sectioning of right ILN, 2) 5% lidocaine given orally	(1) Right unilateral vocal cord paralysis, (2) oropharyngeal anesthesia	Barometric plethysmography EMG	Swallow frequency and swallowing characteristics based on ventilation did not change following unilateral laryngeal paralysis. Swallows during expiration decreased while swallows during inspiration increased following oropharyngeal anesthesia with lidocaine.
**Unilateral cervical vagotomy**	Ouahchi et al. (2017) [19]	Rat/male, 7–11 weeks old, 260–400 g	A right or left cervical vagotomy under the SLN and above the ILN	A right or left laryngeal paralysis	Barometric plethysmography	A right or a left cervical vagotomy does not alter ventilation at rest, but induces during sequential swallowing a decrease in respiratory rate and mean inspiratory flow.
	Ouahchi et al. (2011) [20]	Rats/male, 2–3 months old, 290–350 g	A unilateral vagotomy above the ILN and under the SLN	Bronchial aspirations, unilateral vocal cord paralysis	Barometric plethysmography Laryngo-endoscopic photograph	Following the sectioning of the right vagus nerve, all the rats presented bronchial aspirations and unilateral vocal cord paralysis in the aperture position. Chronic aspiration decreases ventilatory drive and modifies ventilatory pattern during swallowing.

BW, body weight; EMG, electromyograms; ILN, inferior laryngeal nerve; RLN, recurrent laryngeal nerve; SLN, superior laryngeal nerve; VF, vocal fold; VFSS, videofluoroscopic swallow study.

**Table 3:** Characterization of oropharyngeal dysphagia in rodent models of radiation injury.

Injury	Author (Year)	Animal/Sex, Age or BW	Method Inducing Dysphagia	Main Damage	Dysphagia Screening	
					Evaluation	Result
**Radiation injury**	Benedict et al. (2018) [21]	Rat/male, adult	CCRT to head and neck	Tongue dysfunction	Tongue force and mobility measurement	Rats lost weight during CCRT and gained weight following CCRT completion. Tongue strength decreased at 2 weeks and 5 months post-CCRT. Tongue displacement increased only at 5 months post-CCRT.
	King et al. (2020) [22]	Rat/male, 8.5–9 months old, 450–500 g	Bilateral cyroinjuries to the belly of the mylohyoid muscle	Prominent inflammation and necrosis, fibrosis	Histology VFSS and lick testing EMG	Fibrosis was confirmed in the mylohyoid at 2-weeks post-injury. One-week after injury, bolus size decreased, swallowing rate reduced, and licking patterns were altered. Immediately post-injury, there was a significant depression in mylohyoid and thyropharyngeus EMG amplitudes during swallowing.
	Russell and Connor (2014) [23]	Rat/male, 9 and 32 months	Delivery of two fractions of 11 Gy	Radiation injury to tongue muscle	Recording of tongue muscle contractile properties	Radiation was associated with a significant decrease in tongue force production and reduced speed of tongue muscle contraction in both young adult and old groups. Radiation treatment did not exacerbate atrophic changes observed with aging or lead to additional fibrosis formation in the genioglossus muscle.

BW, body weight; CCRT, concurrent chemoradiation therapy; EMG, electromyograms; VFSS, videofluoroscopic swallow study.

**Table 4:** Characterization of oropharyngeal dysphagia in rodent model of other injury.

Injury	Author (Year)	Animal/Sex, Age or BW	Method Inducing Dysphagia	Main Damage	Dysphagia Screening	
					Evaluation	Result
**Gestational retinoid exposure**	Holson et al. (2000) [24]	Rat/pups	Gestational exposure to all-trans-retinoic acid daily on GDs 11 through to 13	Impairment in swallowing	Behavioral response to milk infusion	Almost no RA-exposed neonates were able to swallow milk infused into the oral cavity. In such cases the milk simply dribbled out of the mouth, while the stomach was found to be empty at autopsy.

BW, body weight; GD, gestational day; RA, retinoic acid.

## Data Availability

Not applicable.
